# Action of Sulphate of Quinine on the Spleen

**Published:** 1847-04

**Authors:** 


*Action of Sulphate of Quinine on the Spleen.*—Dr. Pagis, interne of the hospitals at Paris, having undertaken a series of experiments for the purpose of ascertaining the action of sulphate of quinine on the spleen, publishes, in the Gazette des Hôpitaux, the results of his researches.

On a middle-sized dog the spleen was uncovered by two incisions perpendicular to each other. The transverse diameter of the viscus measured twenty centimetres, and the longitudinal six. The jugular vein was opened, and twenty-three grammes of alcoolat of quinine were injected; instantaneously the spleen diminished in every direction, its surface became rough and wrinkled, and its diameters were reduced to fourteen centimetres by five.

On another animal the experiments were repeated, with a view of comparing the results of several injections: with water they were negative, with alcohol the spleen was very slightly corrugated, but with the solution of quinine the viscus contracted instantaneously in the most evident manner.—*Med. Times*, December 26, 1846.

